# Immunohistochemical examination of the uteroplacental interface of cows on days 21, 31, 40, and 67 of gestation

**DOI:** 10.1530/REP-23-0444

**Published:** 2024-01-29

**Authors:** Heewon Seo, Gabriela D Melo, Ramiro V Oliveira, Gessica A Franco-Johannsen, Fuller W Bazer, Ky G Pohler, Gregory A Johnson

**Affiliations:** 1 Department of Veterinary Integrative Biosciences, School of Veterinary Medicine and Biomedical Sciences, Texas A&M University, College Station, Texas, USA,; 2 Department of Animal Science, College of Agriculture and Life Sciences, Texas A&M University, College Station, Texas, USA

## Abstract

**In brief:**

Cattle are classified as having synepitheliochorial placentation in which the majority of the uterine luminal epithelial cells remain intact with some luminal epithelial cells fusing with binucleate trophoblast cells to form syncytial trinucleate cells. This study suggests the possibility that, for a limited and as yet undefined period of gestation, the majority of luminal epithelial cells are eliminated and replaced by trophoblast cells that express pregnancy-associated glycoproteins.

**Abstract:**

What we understand about the early stages of placentation in cattle is based on an elegant series of electron microscopic images that provide exquisite detail but limited appreciation for the microanatomy across the uteroplacental interface. In order to achieve a global perspective on the histology of bovine placentation during critical early stages of gestation, i.e., days 21, 31, 40, and 67, we performed immunohistochemistry to detect cell-specific expression of pregnancy-associated glycoprotein (PAG), cytokeratin, epithelial (E)-cadherin, and serine hydroxymethyltransferase 2 (SHMT2) at the intact uteroplacental interface. Key findings from the immunohistochemical analyses are that there are: (i) PAG-positive cells with a single nucleus within the uterine luminal epithelial (LE) cells; (ii) PAG-positive cells with two nuclei in the LE; (iii) PAG-positive syncytial cells with more than three nuclei in the LE; (iv) LE cells that are dissociated from one another and from the basement membrane in regions of syncytialization within the LE layer; (v) replacement of the mononuclear LE with a multilayer thick population of PAG-positive cells invading into the uterine stroma of caruncles but not into the stroma of intercaruncular endometrium; and (vi) PAG-, E-cadherin-, and SHMT2-positive mononuclear cells at the leading edge of developing cotyledonary villi that eventually represent the majority of the epithelial surface separating caruncular stroma from cotyledonary stroma. Finally, the uteroplacental interface of ruminants is not always uniform across a single cross section of a site of placentation, which allows different conclusions to be made depending on the part of the uteroplacental interface being examined.

## Introduction

**Figure 1:**
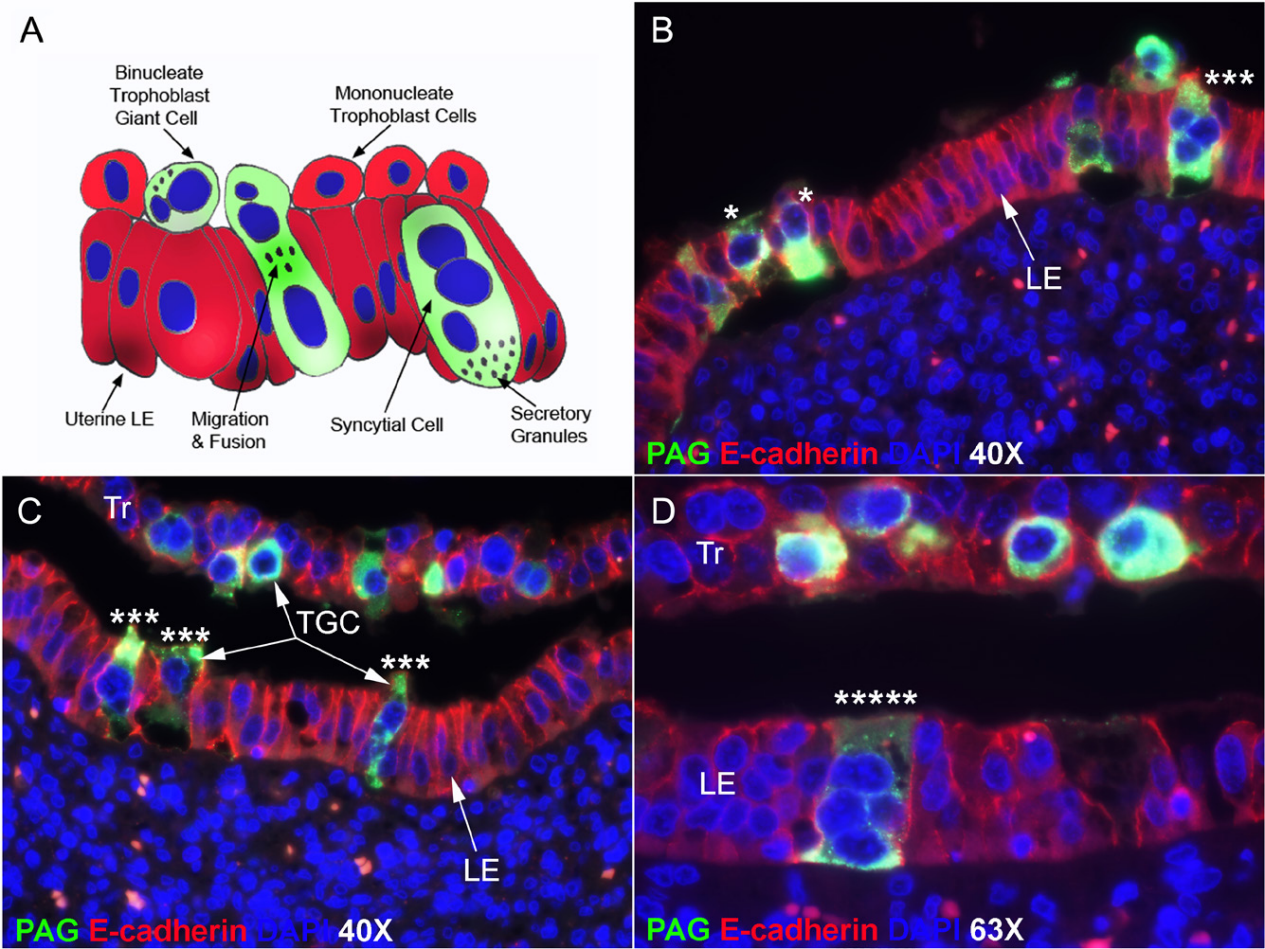
A. A diagram depicting conventional wisdom for early syncytialization of cells in placentae of cattle. Syncytialization is limited to binucleate trophoblast giant cells (TGCs) in the trophoblast layer and trinucleate syncytial cells in the uterine luminal epithelial (LE) layer. B–D. Immunofluorescence staining for PAG (green, stains TGCs and syncytial cells) and E-cadherin (red, stains mononuclear trophoblast (Tr)) within the trophoblast layer and uterine LE on day 21 (B, C) and day 31 (D) of pregnancy. Nuclei are stained blue with DAPI for histological reference. Asterisks indicate TGCs that have at least one (⁎), what appear to be three (⁎⁎⁎), or more than three (⁎⁎⁎⁎⁎) nuclei present within the uterine LE layer. The width of field for B and C is 224 μm and for D is 142 μm.

Two morphologically and functionally distinct trophoblast cell types, mononuclear trophoblast cells and binucleate trophoblast giant cells (TGCs), have been recognized in the trophoblast of the bovine placenta (Wimsatt 1951, Greenstein *et al.* 1958, Wooding 1982*a*,*b*, Hoffman & Wooding 1993). The mononucleate cells constitute the majority of the trophoblast cells in elongating conceptuses (embryo and associated placental membranes). They are responsible for the synthesis and secretion of interferon tau as the pregnancy recognition signal. They are also involved in the attachment of the conceptus to the uterine luminal epithelium (LE) for implantation (Guillomot & Guay 1982, Thatcher *et al.* 1989). TGCs begin to differentiate from the mononuclear trophoblast cells between days 17 and 19 of gestation (Greenstein *et al.* 1958). It has been proposed that TGCs arise through consecutive nuclear divisions without cytokinesis, also termed mitotic polyploidy (Wimsatt 1951, Wooding 1984, Hoffman & Wooding 1993, Klisch *et al.* 1999, Black *et al.* 2010). TGCs comprise 15–20% of the trophoblast cells during the apposition and attachment phases of implantation, and their numbers within the chorionic epithelium remain relatively constant throughout gestation (Wooding 1992, 2022). TGCs do not proliferate; however, an extensive network of rough endoplasmic reticulum and Golgi is responsible for the accumulation of secretory granules as TGCs mature, giving rise to the secretion of placental lactogens, pregnancy-associated glycoproteins (PAG), and prolactin-related proteins among other factors (Fig. 1A) (Duello *et al.* 1986, Wallace *et al.* 2015). After firm attachment of the trophoblast to the uterine LE by days 20–22 of pregnancy, TGCs migrate through the microvillar junctions between the mononucleate trophoblast cells and uterine LE and then fuse with individual uterine LE cells to form trinucleate fetal–maternal syncytial cells (Fig. 1A) (Wooding 2022). The trinucleate cells maintain direct physical apposition to the uterine LE, and their secretory granules are transported across the cytoplasm to the cell surface facing the uterine LE, where they can be released toward the uterine vasculature (Fig. 1A) (Wooding 2022). Indeed, concentrations of PAG in serum can be used to reliably assess TGC development and diagnose pregnancy in cows (Sasser *et al.* 1986, Reese *et al.* 2018).

The gross anatomic appearance of the bovine placenta is dominated by 100–140 focal villous aggregations termed cotyledons (Hradecky *et al.* 1988, Haeger *et al.* 2016). These cotyledons develop and attach to preformed endometrial caruncles to form discrete highly vascularized structures called placentomes, which provide for exchange of oxygen and CO_2_, as well as hemotrophic nutrition in the form of sugars, amino acids, and fatty acids to support growth and development of the fetus (Ferrell *et al.* 1983, Haeger *et al.* 2016). Placentomes are composed of highly branched placental chorioallantoic villi, termed cotyledons, which grow rapidly and interdigitate with maternal aglandular endometrial crypts, termed caruncles. The mechanisms by which cotyledons invade/interdigitate with caruncles are poorly understood. Comprehensive hematoxylin and eosin (H&E) staining, immunohistochemistry, and electron microscopic (EM) studies have established that the chorioallantois of mature placentomes of cows on day 270 of pregnancy is composed of three morphologically and functionally distinct cell types: (i) the mononucleate trophoblast cells; (ii) the binucleate TGCs; and (iii) the trinucleate fetal–maternal hybrid cells (Wimsatt 1951, Wooding 1982*a*, Hoffman & Wooding 1993, Haeger *et al.* 2016). Much remains to be understood about the molecular, cellular, histological, and physiological events that lead to the development of these cell types and their functions within placentomes in cows, as highlighted in a recent report of the identification of five different trophoblast cell types in the cotyledonary and intercotyledonary areas of bovine placentae from day 195 of pregnancy (Davenport *et al.* 2023). However, *in vitro* models are currently being utilized to investigate this complex physiology (Bridger *et al.* 2007, Haeger *et al.* 2011, 2016).

On average, 8–12% of beef females experience pregnancy loss after being diagnosed as pregnant, with that percentage being even higher for high-producing dairy cows. The time of those losses corresponds to periods when there are as yet unclear developmental changes taking place in the placenta and its interaction with the uterus. Inadequate placentation is a potential explanation for embryonic mortality after day 28 of gestation, since placentome formation occurs between days 25 and 50 of gestation in cattle. Importantly, circulating concentrations of PAG are low on day 28 in cows predicted to experience late embryonic mortality, and it is therefore likely that the number of TGCs in the trophoblast of conceptuses predicted to die is reduced (Pohler *et al.* 2016*a*,*b*). What we understand about these stages of placentation in cattle has been illustrated by an elegant series of EM images (Wathes & Wooding 1980, Wooding & Burton 2008). EMs from day 20 of gestation indicate that binucleate cells (BNCs) in the trophoblast tissue layer migrate to the microvillar junction between the trophoblast and uterine LE and that TGCs fuse with LE cells to form trinucleate cells that secrete granules toward the vasculature of the endometrial stroma (Wathes & Wooding 1980). EMs from days 28 and 49 of gestation indicate that TGCs continue to be present in the trophoblast layer, and most LE cells are cuboidal and contain large lipid droplets, and only the pyknotic remnants of the original columnar LE cells remain (Wathes & Wooding 1980). EMs from day 130 of gestation indicate that TGCs migrate from the trophoblast layer into the LE layer and fuse with LE cells to form trinucleate cells that may release granules close to blood vessels in the endometrial stroma before dying and being reabsorbed (Wooding & Burton 2008).

EMs provide exquisite detail but without appreciation for the global microanatomy present across the uteroplacental interface. In order to achieve a global perspective of the histology of bovine placentation during critical early stages of gestation, specifically days 21, 31, 40, and 67, we performed immunohistochemistry to characterize cell-specific and temporal changes in expression of PAG, cytokeratin, epithelial (E)-cadherin, and serine hydroxymethyltransferase 2 (SHMT2) at the intact uteroplacental interface.

## Materials and methods

### Animals and tissue preparation

All animal experimental procedures were approved by the Texas A&M University Institutional Animal and Care and Use Committee. All multiparous, crossbred beef cows were maintained on pastures and had access to water and mineral salt *ad libitum*. Cows were subjected to a 7-day CO-synch plus a control internal drug release fixed-time artificial insemination (CIDR FTAI) protocol: CIDR containing 1.38 g of progesterone (CIDR; Zoetis, New York, NY, USA) and 100 μg (i.m.) of gonadotropin-releasing hormone (GnRH; 2 mL of cystorelin; Merial, Duluth, GA, USA) on day 9, 25 mg (i.m.) of prostaglandin F2α (PGF2α; 5 mL of lutalyse; Zoetis) and CIDR withdrawal on day 2, and a second injection of GnRH (2 mL of cystorelin; Merial) ~66 h after PGF2α and FTAI to a single sire on day 0.

Cows were hysterectomized on days 21 (*n* = 3), 31 (*n* = 3), 40 (*n* = 6), or 67 (*n* = 3), and several 1–1.5 -cm sections of uterine wall and associated placenta from different regions of each uterine horn were fixed in fresh 4% paraformaldehyde in PBS (pH 7.2), changed to 70% ethanol after 24 h, and then dehydrated and embedded in Paraplast-Plus (Oxford Labware, St. Louis, MO, USA).

### Immunofluorescence analyses

Placenta-associated glycoproteins, E-cadherin, cytokeratin, solute carrier family 2 member 1 (SLC2A1), and SHMT2 proteins were localized within the uteroplacental interface of paraffin-embedded samples from days 21, 31, 40, and 67 of pregnancy cows using immunofluorescence microscopy, as described previously (Seo *et al.* 2019). Uteroplacental tissues (at least three slides for caruncular/intercaruncular and placentomal/interplacentomal regions/cow) were sectioned at 5 μm, deparaffinized in xylene, and rehydrated through successive baths of 100% ethanol, 95% ethanol, 70% ethanol, and water. Antigen retrieval was performed using either boiling citrate (E-cadherin, cytokeratin, SLC2A1, and SHMT2) or protease (PAG, E-cadherin, and cytokeratin). Sections were then blocked in 10% normal goat serum for 1 h at room temperature. The tissue sections were then incubated overnight at 4 °C with the following primary antibodies: rabbit anti-PAG polyclonal antibody (kindly provided by Jonathan A. Green, University of Missouri-Columbia, Columbia, MO, 1:100 dilution in antibody dilution buffer), rabbit anti-SHMT2 polyclonal antibody (Sigma-Aldrich, HPA020543; 1:100 dilution), mouse anti-E-cadherin monoclonal antibody (BD Biosciences, San Jose, CA, USA, 610182; 1:200 dilution), and mouse anti-cytokeratin monoclonal antibody (Sigma-Aldrich, C-6909; 1:500 dilution). For controls, tissue sections were also incubated in normal rabbit (EMD Millipore, 12-370) or mouse (EMD Millipore, 12-371) IgGs at concentrations equal to those for the primary antibodies. Immunoreactive proteins were detected using the appropriate Alexa Fluor 488- or Alexa Fluor 594-conjugated secondary antibodies (Life Technologies) for 1 h at room temperature at a dilution of 1:250. Tissue sections were then washed three times for 5 min/wash in PBS. Slides were counterstained with Prolong Gold Antifade reagent containing DAPI (Life Technologies) and coverslipped. Images were taken using an Axioplan 2 microscope (Carl Zeiss) interfaced with an Axioplan HR digital camera.

## Results

### Evidence suggesting binucleate TGCs are not the only cells that migrate into the uterine LE and that syncytialization within the uterine LE is not limited to the formation of trinucleate cells in cows

**Figure 2:**
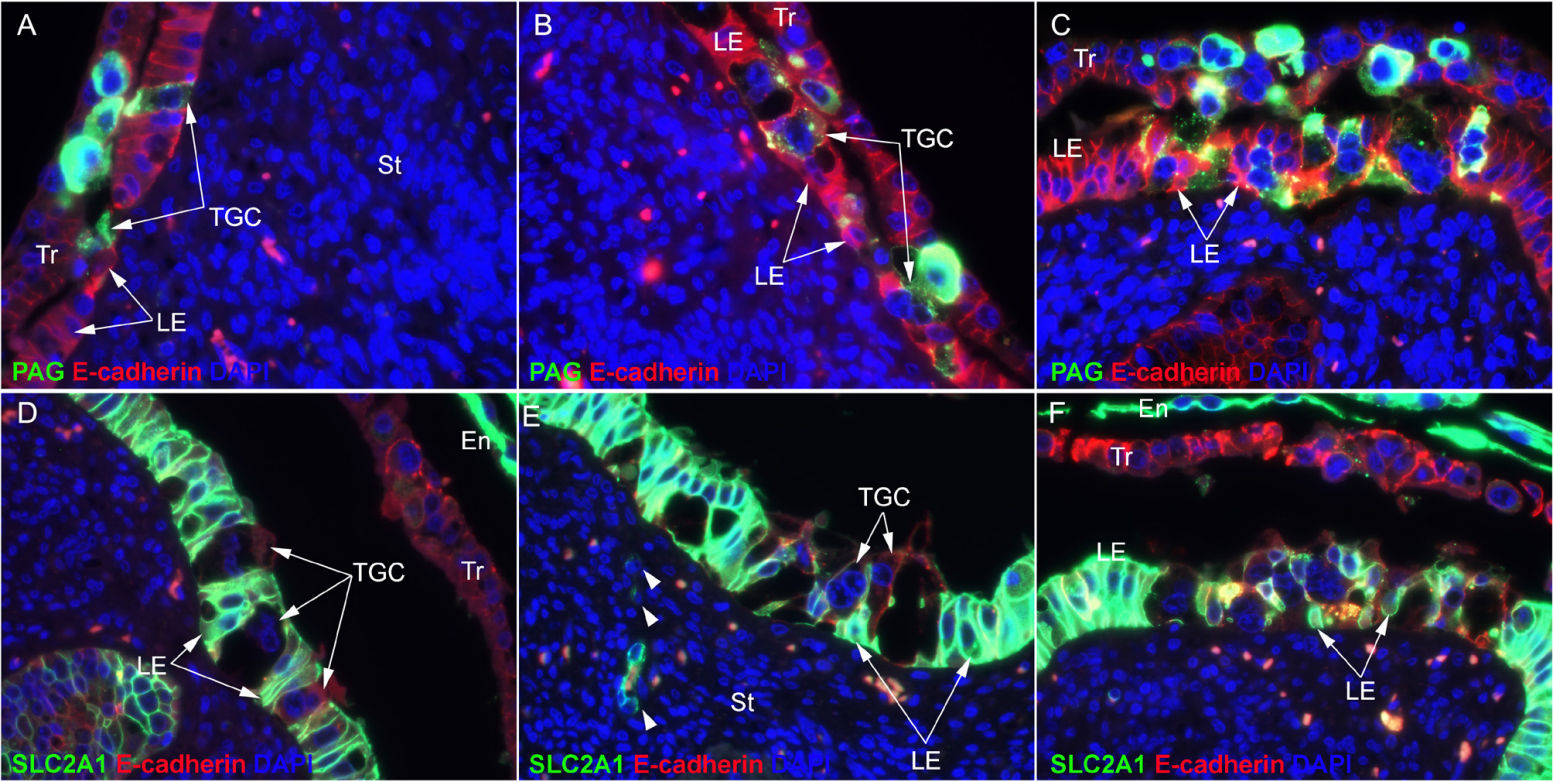
A–C. Immunofluorescence staining for PAG (green, stains trophoblast giant cells (TGCs)) and E-cadherin (red, stains mononuclear trophoblast (Tr) and uterine luminal epithelium (LE)) within the uteroplacental interface on day 21 of gestation. PAG-positive cells are present within the LE layer. D–F. Immunofluorescence staining for solute carrier family 2 member 1 (SLC2A1; green, stains uterine LE and conceptus endoderm (En)] and E-cadherin (red, stains all mononuclear Tr cells within the trophoblast layer and some TGCs within the LE) within the uteroplacental interface on day 21 of pregnancy. D and E. TGCs within the LE layer, some of which appear to have two nuclei. E. Presence of SLC2A1-positive LE cells within the uterine stroma (arrowheads). F. LE cells have dissociated from the neighboring epithelial cells and the basement membrane. Nuclei are stained blue with DAPI for histological reference. The width of field for all panels is 224 μm.

Both mononuclear trophoblast cells and uterine LE cells stained positively for E-cadherin, while PAG protein was localized to binucleate TGCs in the conceptus trophoblast layer and to trinucleate cells within the uterine LE on day 21 of gestation using immunofluorescence microscopy (Fig. 1B). However, what appear to be mononuclear PAG-positive cells were identified within the uterine LE layer. It should be noted that the number of nuclei in these cells could not be confirmed using immunohistochemistry (Fig. 1C). In addition, PAG-positive syncytial cells with more than three nuclei (Fig. 1D) and cells with two nuclei (Fig. 2C, D, and E) were observed in the uterine LE layer on day 21 of pregnancy.

### The arrival of PAG-positive TGCs within the uterine LE layer results in extensive destabilization of the mononuclear LE barrier to the underlying uterine stroma in cows

Figure 2 depicts the uteroplacental interface on day 21 of pregnancy having PAG-positive cells within the uterine LE layer (Fig. 2A, B, andC), and disruption of the integrity of the uterine LE monolayer is evident when visualizing immunofluorescence staining for SLC2A1, a glucose transporter (Fig. 2D, E, and F). It appears that TGC migration on day 21 of gestation results in greater alteration of the uterine LE than one would expect based on conventional wisdom as summarized in Fig. 1A. Many of the LE cells are dissociated from one another and from the basement membrane providing greater exposure of the stromal vasculature to TGCs than previously appreciated (Fig. 2). Further, SLC2A1-positive cells that may represent uterine LE are present within the stroma just below the uterine LE into which TGCs are invading (Fig. 2E).

### Large numbers of TGCs invade into limited regions of the uterine LE as the conceptus attaches to the uterine wall on day 21 of gestation in cows

**Figure 3:**
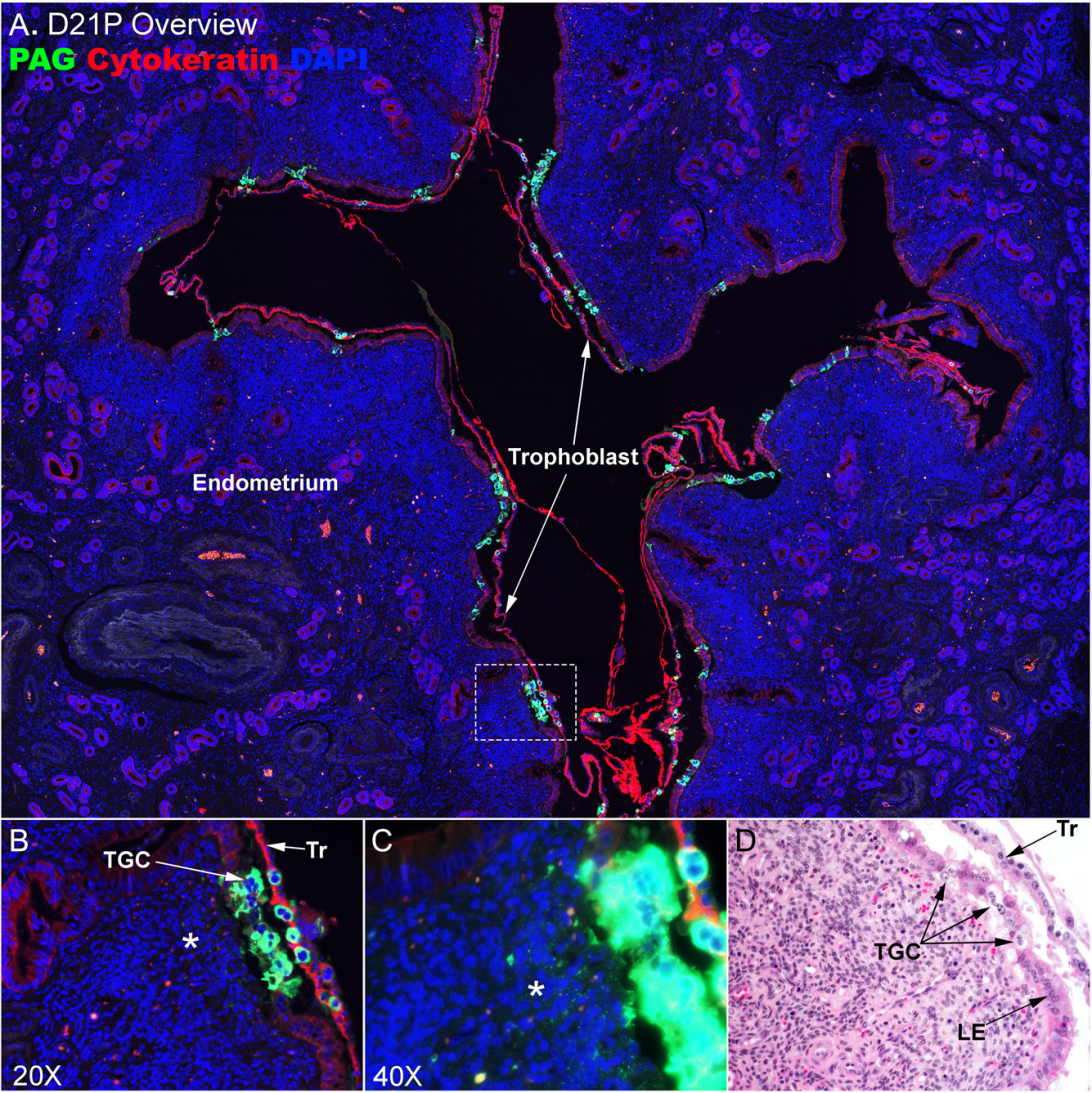
The uteroplacental interface on day 21 of bovine pregnancy. (A–C) Immunofluorescence staining for PAG (green, stains trophoblast giant cells (TGCs)) and cytokeratin (red, stains mononucleate trophoblast (Tr) and uterine luminal epithelium (LE) at an implantation site on day (D) 21 of pregnancy (P). Nuclei are stained blue with DAPI for histological reference. (A) An overview of an implantation site. (B and C). Higher magnification images of the region surrounded by the dotted lined box in A. The image in panel C has been overexposed so that positive immunostaining for PAG can be seen in the uterine stroma. There is migration of TGCs into limited regions of the uterine LE cell layer. Asterisks (⁎) indicate positive immunostaining for PAG in the uterine stroma. D. H&E staining at an implantation site illustrates the presence of TGCs within the uterine LE layer. The width of field for A is 4120 μm, B 448 μm, C 224 μm, and D 369 μm.

On day 21 of pregnancy, the majority of the uteroplacental interface remains epitheliochorial, with only small areas of the uteroplacental interface having PAG-positive TGCs within the uterine LE (Fig. 3A). Invasion of TGCs into those regions is, however, very robust (Fig. 3B). In addition, PAG immunostaining that is evident within the uterine stroma (Fig. 3C) likely represents granules released by PAG-positive cells within the LE (Fig. 3D).

### The mononuclear LE barrier is lost along extensive expanses of the uteroplacental interface on day 31 of gestation in cows

**Figure 4:**
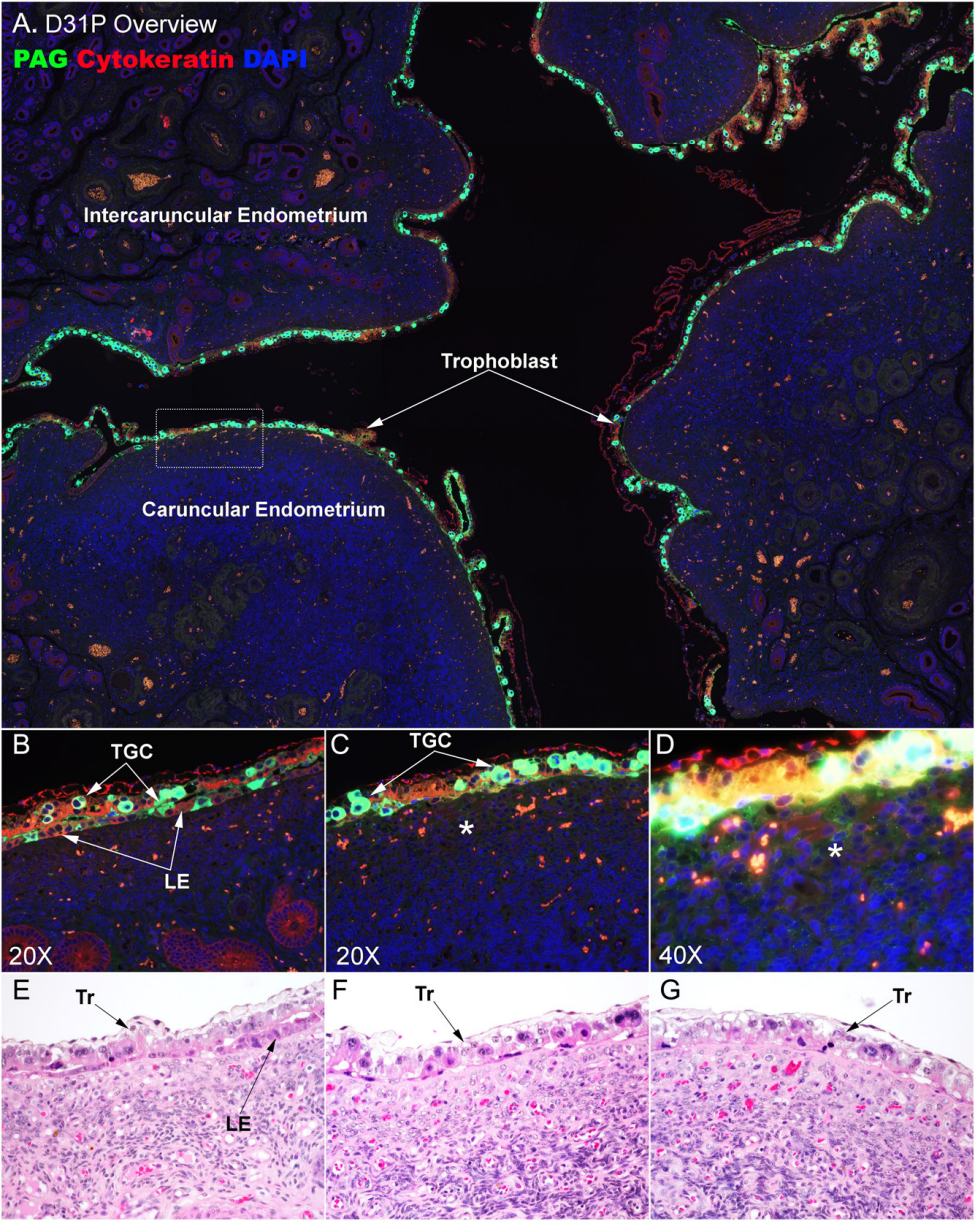
The uteroplacental interface on day 31 of bovine pregnancy. (A–D) Immunofluorescence staining for PAG (green, stains trophoblast giant cells (TGCs)) and cytokeratin (red, stains mononucleate trophoblast (Tr) and uterine luminal epithelium (LE)) at an implantation site on day (D) 31 of pregnancy (P). Nuclei are stained blue with DAPI for histological reference. (A) An overview of an implantation site. B. A region of the uteroplacental interface where the mononuclear LE sheet is maintained. (C and D) Higher magnification images of the region surrounded by the dotted lined box in A. The image in panel D has been overexposed to illustrate PAG immunostaining in the uterine stroma (⁎). (E–G) Images of H&E staining with the LE monolayer mostly maintained in E, extensively replaced in F, and completely gone in G. Different stages of invasion of LE cells by TGCs were seen in immunofluorescence staining (B and C) and H&E staining (E–G). The width of field for A is 4120 μm, B and C 448 μm, D 224 μm, and E and F is 369 μm.

On day 31 of pregnancy, the majority of the uteroplacental interface experiences invasion of TGC into the uterine LE, and large areas of the LE are absent from the interface (Fig. 4A). Figures 4B and E illustrate that some LE cells are present in some regions of the uteroplacental interface, and these LE cells are low cuboidal in shape. Figures 4C and F illustrate a more extensive loss of uterine LE and its replacement with PAG-positive TGCs. In some regions of the uterine LE, no intact LE cells remain to act as a barrier between the PAG-positive TGCs and the underlying uterine stroma (Fig. 4G), and the uteroplacental interface appears syndesmochorial in these regions. Figure 4D shows that PAG-positive cells are present in the stroma just below regions devoid of LE that has been replaced with TGC in the uterine LE layer.

### The uteroplacental interface on days 40 and 67 of gestation in cows. PAG-positive TGCs begin to invade into the caruncular stroma, and E-cadherin/SHMT2-positive mononuclear cells are present at the leading front of invasion into the caruncular stroma to become the epithelium separating caruncular stroma from cotyledonary stroma in placentomes

**Figure 5:**
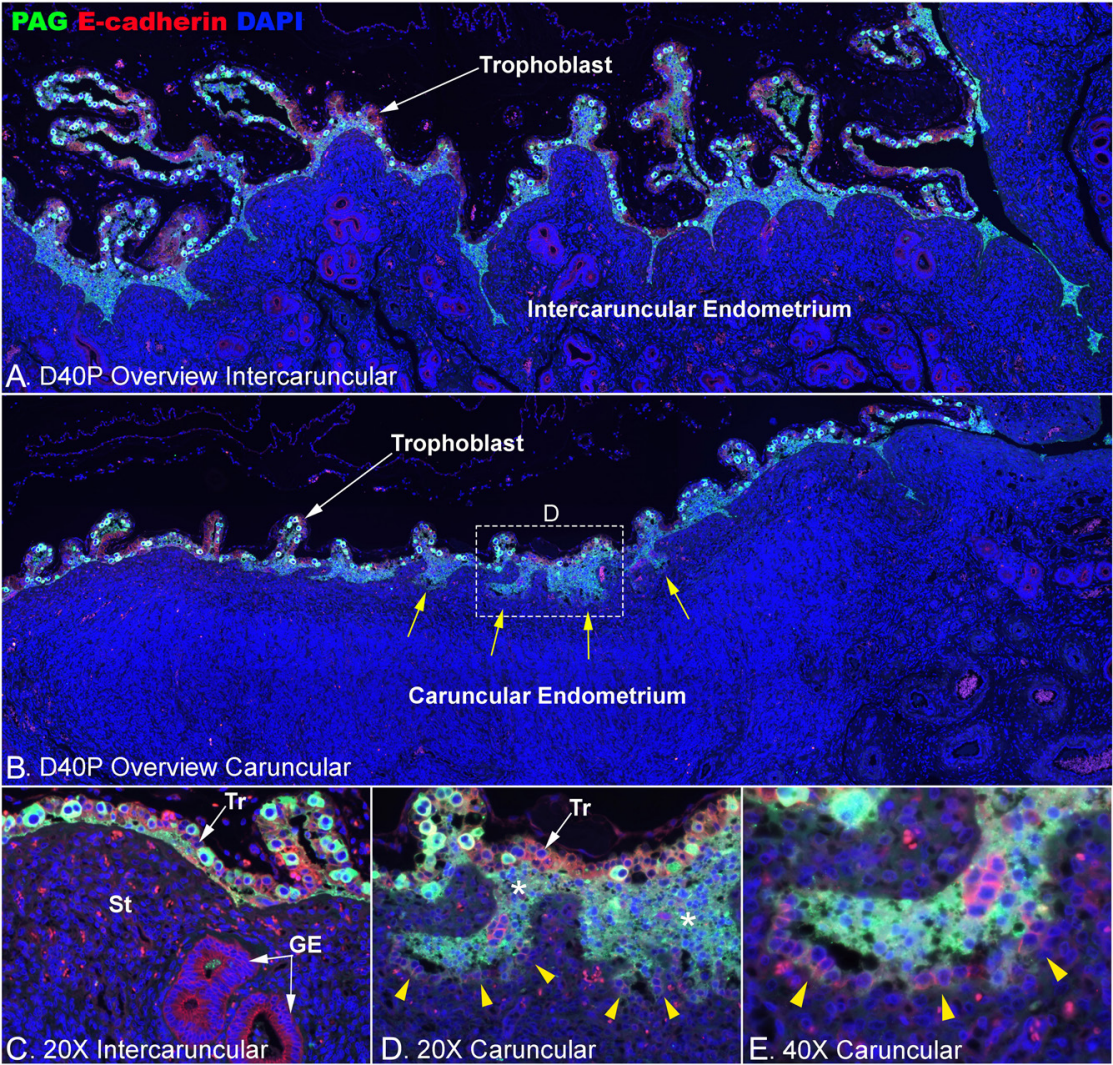
The uteroplacental interface on day 40 of bovine pregnancy. Immunofluorescence staining for PAG (green, stains trophoblast giant cells (TGCs)) and E-cadherin (red, stains mononucleate trophoblast (Tr) and uterine luminal epithelium (LE) and glandular epithelium (GE)) at an implantation site on day (D) 40 of pregnancy (P). Nuclei are stained blue with DAPI for histological reference. A. Intercaruncular uteroplacental interface. B. Caruncular uteroplacental interface. C. A higher magnification image of a region of intercaruncular uteroplacental interface. D and E. Higher magnification images of the region surrounded by the dotted lined box in B. Almost all of the uterine LE cells are lost and replaced by PAG-positive cells over the entire endometrial surface at implantation sites, and PAG-positive cells appear to invade into the caruncular endometrium (⁎ in D). (D andE) Along with TGCs, E-cadherin-positive mononuclear cells also migrate at the invasion front. Arrowheads illustrate the mononuclear cells at the leading edge of the newly forming cotyledonary villi. The width of field for A and B is 4120 µm, C and D 448 μm, and E 224 μm. St, stroma.

On day 40 of gestation, few mononuclear uterine LE cells remain at the uteroplacental interface at sites of implantation in cattle (Fig. 5). In their place, there is a thick multilayered population of PAG-positive cells invading into the uterine stroma of caruncles (Fig. 5B, D, and E) but not into the stroma of intercaruncular endometrium (Fig. 5A and C). Further, the cells at the leading front of invasion are mononuclear and express E-cadherin (weak PAG immunostaining is also evident), suggesting that they are either remaining LE cells or that they are mononuclear trophoblast cells that have migrated to this region from the trophoblast cell layer (Fig. 5D and E). No mononuclear E-cadherin-positive cells are found adjacent to the stroma in intercaruncular regions associated with placentation, and E-cadherin-positive mononuclear cells are limited to caruncular regions where TGCs are invading into the stroma, presumably as precursors for the formation of cotyledonary villi (Fig. 5).

**Figure 6:**
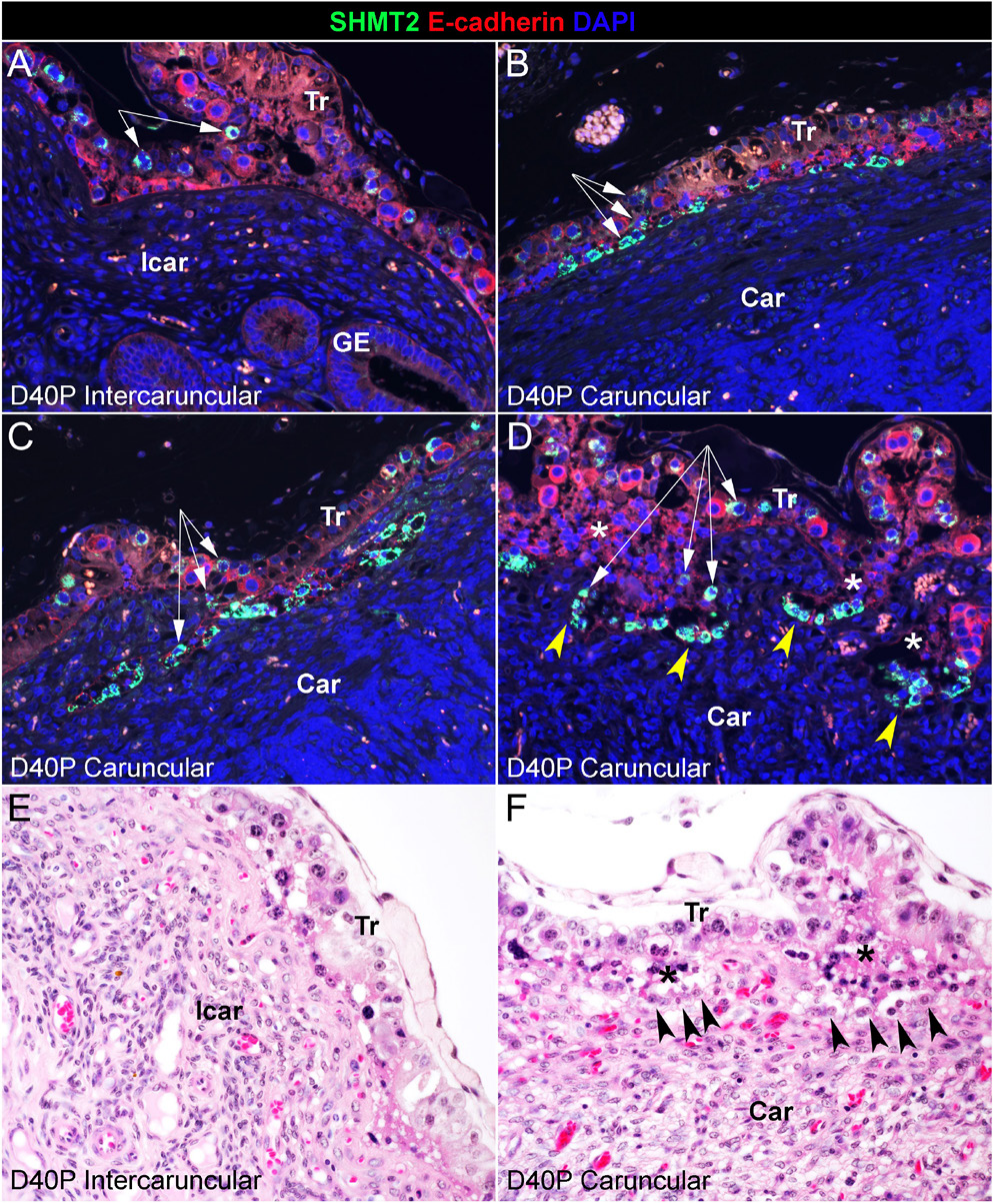
A–D. Immunofluorescence staining for serine hydroxymethyltransferase 2 (SHMT2; green, stains some trophoblast cells in the trophoblast layer (white arrows) and all the mononuclear cells at the leading front of placental invasion into the caruncular stroma (yellow arrowheads)), and E-cadherin (red). E and F. H&E staining of day 40 bovine implantation sites illustrates invasion of the caruncular stroma (⁎) and mononuclear cells at the leading edge of invasion into the caruncular stroma (arrowheads). The width of field for A–C is 448 μm and for E and F is 369 μm. Car, caruncular stroma; D, day; GE, glandular epithelium; Icar, intercaruncular stroma; P, pregnancy; Tr, trophoblast.

**Figure 7:**
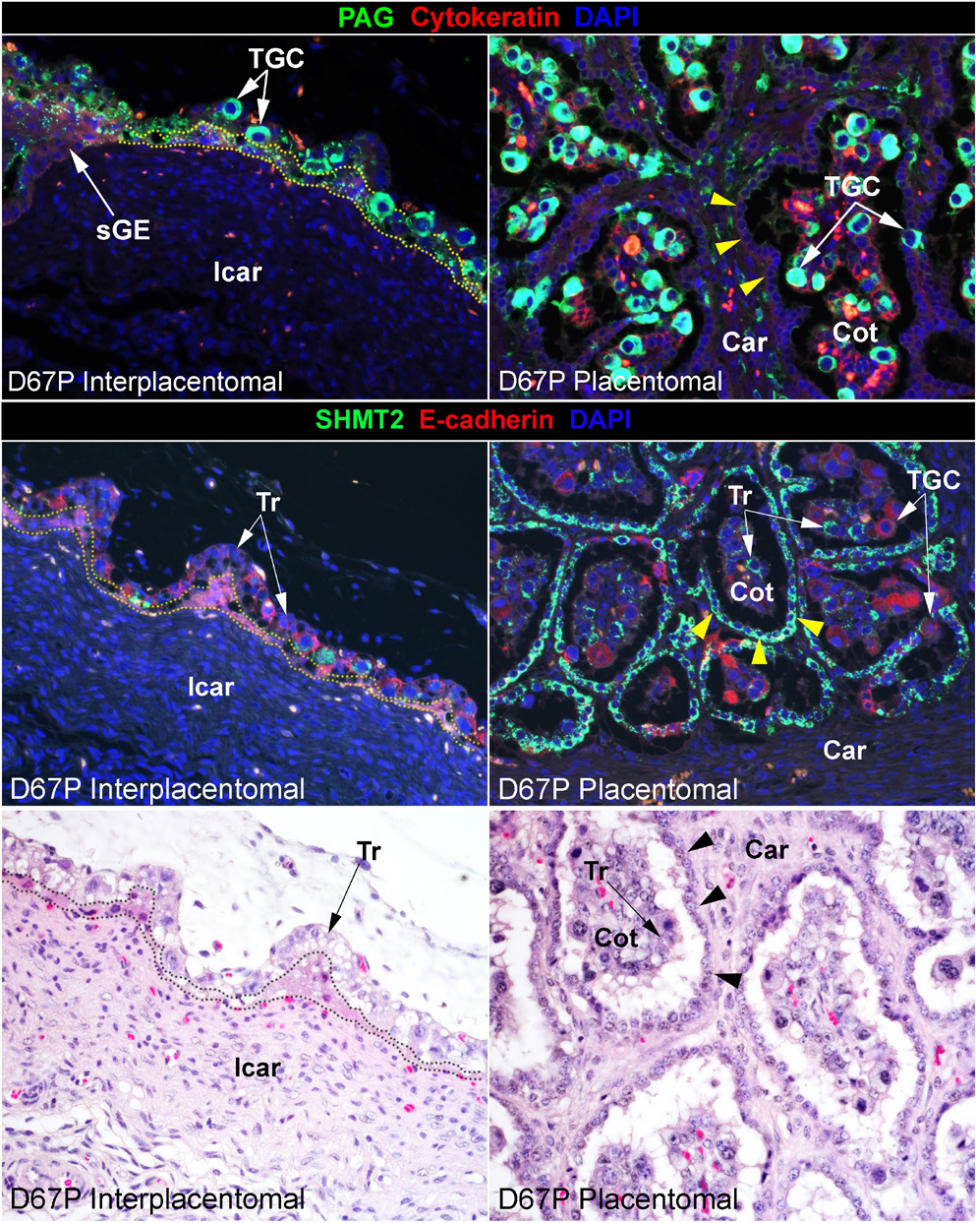
The uteroplacental interface on day 67 of bovine pregnancy. Top: Immunofluorescence staining for PAG (green, stains trophoblast giant cells (TGCs)) and cytokeratin (red, stains mononucleate trophoblast (Tr), uterine luminal epithelium (LE), and shallow glandular epithelium (sGE)) at an implantation site on day (D) 67 of pregnancy (P). Middle: Immunofluorescence staining for serine hydroxymethyltransferase 2 (SHMT2; green, stains mononuclear cells lining the caruncular mesenchyme) and E-cadherin (red, stains mononucleate trophoblast (Tr), and some syncytial trophoblasts). The SHMT2-positive mononucleate cells have expanded to become the epithelial surface separating the caruncular stroma from the cotyledonary stroma within the placentome (yellow and black arrowheads). Bottom: H&E staining. Car, caruncular stroma; Cot, cotyledon; Icar, intercaruncular stroma. The width of field for the images of immunofluorescence staining is 448 μm and for the images of H&E staining is 369 μm.

The SHMT2 gene encodes for an enzyme within the one-carbon metabolic pathway that is activated in proliferating and migrating trophoblast cells at the uteroplacental interface of pigs and in elongating conceptuses of sheep (Johnson *et al.* 2022, Halloran *et al.* 2023). SHMT2 is expressed by some trophoblast cells in the trophoblast layer and by all of the E-cadherin-positive mononuclear cells (Fig. 5) at the leading front of invading TGCs on day 40 of gestation (Fig. 6). The pattern of expression of SHMT2 across the uteroplacental interface suggests possible migration of SHMT2-positive trophoblast cells from the trophoblast layer to the surface of the caruncular stroma lacking the LE layer (Fig. 6B) into the caruncular stroma (Fig. 6C) and onto the leading edge of newly forming cotyledonary villi (Fig. 6D). In intercaruncular regions of the uteroplacental interface, there is no evidence of invasion into the intercaruncular stroma, and immunostaining for SHMT2 is limited to a few cells in the trophoblast layer (Fig. 6A). The results of H&E staining support a lack of invasion of cells into intercaruncular stroma (Fig. 6E) but confirm invasion of mononuclear cells into the caruncular stroma (Fig. 6F). By day 67 of gestation, the SHMT2-positive mononucleate cells have expanded to form the majority (there is also a smaller population of PAG-positive TGCs) of the epithelial surface separating the caruncular stroma from cotyledonary stroma, and PAG-positive TGCs are predominate in the cotyledonary trophoblast layer (Fig. 7). It is noteworthy that at the surface of the interplacentomal stroma, there is no accumulation or invasion by SHMT2-positive cells (Fig. 7).

## Discussion

Through an elegant series of EMs, it has been convincingly argued that during bovine placentation, migrating binucleate cells in the trophoblast layer disrupt the microvillar interdigitation between the apical surfaces of mononucleate trophoblast cells and uterine LE cells allowing direct contact and fusion of the binucleate cell with the apical surface of the LE cell to form a trinucleate cell. This trinucleate cell then establishes lateral connections with adjacent LE cells, connects to the basement membrane of the LE layer, and maintains an apposition with the apical surface of cells in the trophoblast layer. The result is insertion of trophoblast protoplasm into the uterine LE layer and exposure of this protoplasm to the underlying uterine stroma while maintaining the structural integrity of the two apposing epithelial trophoblast and LE sheets (Wooding 2022) (Fig. 1A). As such, the primarily epitheliochorial nature of bovine placentation is a stable feature throughout pregnancy, and the term ‘syn’ in synepitheliochorial applies to the binucleate and trinucleate cells. The results presented in this study do not refute that this process takes place but strongly suggest that placental development in the cow is more complex than that described previously based on analyses of EMs. Rather, the stability of the intact apposing epithelial trophoblast and uterine LE is temporally compromised during early gestation, perhaps being a factor in much of the 8–12% pregnancy losses observed in cattle post pregnancy diagnosis due to the timing of these losses and temporal placental remodeling.

Key observations from immunohistochemistry for PAG, cytokeratin, E-cadherin, and SHMT2 on paraformaldehyde-fixed, paraffin-embedded, thin cross sections of the intact uteroplacental interface across the entire wall of bovine uteri from days 21, 31, 40, and 67 include: (i) PAG-positive cells that appear to have a single nucleus within the LE layer; (ii) PAG-positive cells that appear to have two nuclei in the LE layer; (iii) PAG-positive syncytial cells with more than three nuclei in the LE layer; (iv) LE cells that have dissociated from one another and dissociated from the basement membrane in regions of syncytialization within the LE layer; (v) replacement of the mononuclear LE sheet with a multilayer thick population of PAG-positive cells that are invading into the uterine stroma of caruncles but not into the stroma of intercaruncular endometrium; and (vi) PAG-, E-cadherin-, and SHMT2-positive mononuclear cells are at the leading edge of developing cotyledonary villi that are eventually the majority of the epithelial surface separating caruncular stroma from cotyledonary stroma. It is worth noting that the PAG antibody used in these experiments is one that has been well-characterized by our group in several different experiments and physiological states (Green *et al.* 2005, Pohler *et al.* 2016*a*,*b*, Gatea *et al.* 2018, Reese *et al.* 2018). This antibody was specifically raised against early secreted PAGs and most likely is recognizing both modern- and ancient-based PAG proteins (Green *et al.* 2005).

When considering statements 1 through 3 above, 5 µm tissue sections limit the ability to assess the microanatomy across the entire cell. It is possible that what appear to be syncytia containing more than three nuclei in the LE layer are actually trinucleate cells that are very close together and only appear to be fused, although the lack of evidence for plasma membranes separating the cells argues against this. It is also possible that the PAG-positive mononuclear trophoblast cells are binuclear TGCs for which the observed nucleus obscured the detection of the second nucleus. However, the frequency of occurrence of these cells in this study and the even greater frequency of these cells in sheep placentation (Green *et al.* 2021, Seo *et al.* 2023) suggest that some PAG-positive cells are mononuclear. Further, it is possible that the two nuclei in PAG-positive binucleate TGCs within the LE layer obscure a third nucleus, and these cells are actually trinucleate syncytia. However, it is unlikely that the single nuclei observed in the PAG-positive mononuclear cells obscures perception of two other nuclei within the cell. Therefore, these cells are likely either mononucleate or binucleate TGCs, raising the possibilities that mononucleate TGCs invade between LE cells without fusing with the apical membranes of LE cells, binucleate TGCs invade into the LE layer without fusing with an LE cell, and/or mononucleate TGCs in the trophoblast layer fuse with other mononucleate TGCs in the LE layer to form binucleate TGCs. Therefore, for cattle, it is not clear if: (i) only binucleate TGCs are migratory; (ii) TGC migration is only to allow fusion with uterine LE cells; or (iii) syncytialization is limited to the formation of trinucleate cells. The EMs have an even greater limitation in assessing the entire microanatomy of cells. Therefore, in order to better understand the spatial relationships among cells within the uteroplacental interface of cows, multiphoton super-resolution confocal microscopy is desirable because femtosecond-pulsed infrared laser wavelengths allow for high-resolution optical sectioning of tissues two- to four-fold thicker than is possible with confocal microscopy.

Ruminant placentation was initially classified as syndesmochorial based on evidence that uterine LE cells were lost, resulting in direct contact between the trophoblast and the uterine stroma (Grosser 1909, 1927). Subsequently, investigators concluded that uterine LE is incorporated into syncytial plaques and ruminants were classified as having synepitheliochorial placentation (Wooding 1992). However, evidence for erosion of uterine LE and direct exposure of syncytia to the uterine stroma in the intercaruncular regions of placental attachment has been demonstrated in sheep (Seo *et al.* 2019, 2020, 2023, Frank *et al.* 2021) and cattle (Davenport *et al.* 2023, Johnson *et al.* 2023). This is particularly intriguing because, in the mature placenta, these regions of the uteroplacental interface have a true epitheliochorial arrangement between placental and uterine tissues in which the mononuclear LE layer is intact in both species. It is unknown how the uterine LE is regenerated in these regions of placentation, but it has been proposed in sheep that the surviving shallow GE cells begin to proliferate and express integrins that bind to osteopontin (OPN, Secreted Phosphoprotein 1 (SPP1)) to form integrin adhesion complexes that give the cells traction between their cytoskeleton and the extracellular matrix as they migrate along the stromal surface to re-epithelialize the uteroplacental interface (Huttenlocker & Horwitz 2011, Seo *et al.* 2020). In this study, we detected discrete regions of day-21 placentation in which the LE was clearly disrupted by the arrival of PAG-positive TGCs. In those areas, the uterine LE lost the sheet-like epithelial arrangement and appeared to lose its lateral junctional connections and connections to the basement membrane (Figs. 2 and 3). By day 31 of gestation, large but limited expanses of the uterine LE had been lost (Fig. 4), and by day 40 of gestation, no mononuclear LE was evident at the uteroplacental interface of either caruncular or intercaruncular regions of placental attachment (Fig. 5). Where there was once an intact uterine LE, there was by day 40 a thick multilayer population of PAG-positive presumably trophoblast cells, or perhaps syncytia composed of trophoblast and LE (Fig. 5). These results suggest that the bovine placenta may be syndesmochorial for some, as yet to be determined, period of gestation. As previously mentioned, pregnancies that have increased likelihood to undergo pregnancy loss during this time period have decreased circulating concentrations of PAG. These data may provide insights into one potential explanation as to why this might occur. One intriguing hypothesis is that these compromised pregnancies lack the ability to remodel the LE sufficiently enough to allow PAG-positive cells access to the uterine stroma for the delivery of their products into the maternal blood supply.

Placentomes are composed of highly branched placental chorioallantoic villi, termed cotyledons, which grow rapidly and interdigitate with maternal aglandular endometrial crypts, termed caruncles. In cows, the villous ‘tree’ consists of an epithelial surface layer termed the chorionic epithelium or trophoblast and a mesenchymal core that contains branches of allantoic blood vessels. The outer layer of the endometrial crypts consists of an epithelium called the caruncular epithelium, and the interior consists of extensions of the endometrial stroma containing endometrial vasculature (Haeger *et al.* 2016). The mechanisms by which cotyledons invade/interdigitate with caruncles are poorly understood, and this warrants future study. Figure 5 shows the uteroplacental interface at both caruncular and intercaruncular sites of placental attachment on day 40 of gestation. No mononuclear LE cells remain, and the PAG-positive cells that oppose the uterine stroma are not invading into the intercaruncular stroma. In contrast, the caruncular uteroplacental interface exhibits the early signs of cotyledonary invasion into the caruncular stroma to form placentomes. Interestingly, no mononuclear cells were present at the interface between the PAG-positive trophoblast cells and the caruncular stroma, except precisely at the leading invasive front of the newly forming cotyledonary villi (Fig. 5). These mononuclear cells express E-cadherin, PAG, and SHMT2, a protein expressed in the trophoblast but not uterine LE of pigs (Johnson *et al.* 2022). SHMT2-positive cells were also detected scattered throughout the trophoblast layer (Fig. 6A, B, C, and D). Figure 6A illustrates a region of placentation in which there is no placental invasion taking place, and the SHMT2 cells are spaced randomly within the trophoblast layer. Figure 6B also shows no invasion, and the SHMT2 cells are only aligned against the caruncular stroma. In Fig. 6C, the SHMT2 cells are imbedded within the stroma, and in Fig. 6D, these cells are at the leading edge of the cotyledonary invasion with placental PAG-positive placental cells migrating behind them (Fig. 5D and E). These mononuclear SHMT2- and PAG-positive cells may be surviving uterine LE cells because LE cells also express SHMT2 on days 21 and 31 of pregnancy (Supplementary Fig. 1, see section on supplementary materials given at the end of this article), but, if that is the case, they are the only LE cells that remain in either intercaruncular or caruncular regions of placental attachment by day 40 of gestation (Figs. 5 and 6). It is reasonable to hypothesize that these cells are a novel population of invasive trophoblast cells. By day 67 of gestation, these SHMT2-positive mononuclear cells compose the majority of the epithelium separating caruncular stroma from cotyledonary stroma. Further studies to confirm the origin of this epithelium are warranted.

In conclusion, the uteroplacental interface of ruminants is not uniform on any given day of pregnancy. This is clearly illustrated in Fig. 3, showing placentation on day 21 when TGCs are initially invading into the uterine LE, and Fig. 5B showing placentation on day 40 when cotyledons are initially invading into the caruncles as described in a previous study of placentation in sheep (Johnson *et al.* 2023). Different degrees of TGC and cotyledon invasion are observed in different regions of a single cross section of an implantation site. Therefore, different conclusions can be made depending on the section of uteroplacental interface being examined.

## Supplementary materials

Supplemental Figure 1. Immunofluorescence staining for serine hydroxymethyltransferase 2 [SHMT2; green, stains some mononucleate trophoblast cells and trophoblast giant cells (TGCs) in the trophoblast layer, extraembryonic endoderm (En) cells and luminal epithelial (LE) cells] and E-cadherin (red) at the uterine-placental interface on day 20 (A) and day 31 (B). SHMT2 is expressed by mononucleate cells (arrow heads) at the leading front of placental invasion into the caruncular stroma on day 40 (C). The SHMT2-positive mononucleate cells (arrow head) have expanded to become the epithelial surface separating the caruncular stroma from the cotyledonary stroma within the placentome on day 67 (D). The width of field for Panels A-D is 448 μm. Car, caruncular stroma; Tr, trophoblast; D, day; P, pregnancy.

## Declaration of interest

The authors declare that there is no conflict of interest that could be perceived as prejudicing the impartiality of the study reported. G Johnson is on the editorial board of *Reproduction*. G Johnson was not involved in the review or editorial process for this paper, on which he is listed as an author.

## Funding

This work was supported by Agriculture and Food Research Initiative Competitive (grant nos. 2017-67015-26457 and 2021-67015-33675) from the USDA National Institute of Food and Agriculturehttp://dx.doi.org/10.13039/100005825 and Texas A&M Triads for Transformation grants 2018 and 2021.

## Author contribution statement

HS performed the immunofluorescence localization, image capture, and assembly of figures. He also contributed to necropsies and to the interpretation of data and manuscript preparation. GDM, RVO, and GAFJ contributed to daily animal husbandry and to necropsies. FWB contributed to the interpretation of data and manuscript preparation. KGP codirected the study, contributed funding to support the study, the interpretation of data, and manuscript preparation. GAJ codirected the study, contributed funding to support the study, the interpretation of data, assembly of figures, and manuscript preparation.

## References

[bib1] Black SG Arnaud F Palmarini M & Spencer TE 2010 Endogenous retroviruses in trophoblast differentiation and placental development. American Journal of Reproductive Immunology 64 255–264. (10.1111/j.1600-0897.2010.00860.x) 20528833 PMC4198168

[bib2] Bridger PS Haupt S Klisch K Leiser R Tinneberg HR & Pfarrer C 2007 Validation of primary epithelioid cell cultures isolated from bovine placental caruncles and cotyledons. Theriogenology 68 592–603. (10.1016/j.theriogenology.2007.05.046) 17580088

[bib3] Davenport KM Ortega MS Johnson GA Seo H & Spencer TE 2023 Review: implantation and placentation in ruminants. Animal 17(Supplement 1) 100796. (10.1016/j.animal.2023.100796) 37567669

[bib4] Duello TM Byatt JC & Bremel RD 1986 Immunohistochemical localization of placental lactogen in binucleate cells of bovine placentomes. Endocrinology 119 1351–1355. (10.1210/endo-119-3-1351) 3525130

[bib5] Ferrell CL Ford SP Prior RL & Christenson RK 1983 Blood flow, steroid secretion and nutrient uptake of the gravid bovine uterus and fetus. Journal of Animal Science 56 656–667. (10.2527/jas1983.563656x) 6841301

[bib6] Frank JW Steinhauser CB Wang X Bughardt RC Bazer FW & Johnson GA 2021 Loss of ITGB3 in ovine conceptuses decreases conceptus expression of NOS3 and SPP1: implications for the developing placental vasculature†. Biology of Reproduction 104 657–668. (10.1093/biolre/ioaa212) 33232974

[bib7] Gatea AO Smith MF Pohler KG Egen T Pereira MHC Vasconselos JLM Lawrence JC & Green JA 2018 The ability to predict pregnancy loss in cattle with ELISAs that detect pregnancy associated glyucoproteins is antibody dependent. Theriogenology 108 269–276. (10.1016/j.theriogenology.2017.12.021) 29275034

[bib8] Green JA Parks TE Avalle MP Telugu BP McLain AL Peterson AJ McMillan W Mathialagan N Hook RR Xie S, *et al.* 2005 The establishment of an ELISA for the detection of pregnancy-associated glycoproteins (PAGS) in the serum of pregnant cows and heifers. Theriogenology 63 1481–1503. (10.1016/j.theriogenology.2004.07.011) 15725453

[bib9] Green JA Geisert RD Johnson GA & Spencer TE 2021 Implantation and placentation in ruminants. Advances in Anatomy, Embryology and Cell Biology 234 129–154. 34694480 10.1007/978-3-030-77360-1_7

[bib10] Greenstein JS Murray RW & Foley RC 1958 Observations on the morphogenesis and histochemistry of the bovine preattachment placenta between 16 and 33 days of gestation. Anatomical Record 132 321–341. (10.1002/ar.1091320308) 13637407

[bib11] Grosser O 1909 Vergleichende anatomie und Entwicklungsgeschichte der eihaute und der placenta. Braumuller W; Vienna and Leipzig.

[bib12] Grosser O 1927 Fruhentwicklung, Eihautbidung und Placentation des Menschen und der Saugetiere. Munchen: J. F.Bergmann.

[bib13] Guillomot M & Guay P 1982 Ultrastructural features of the cell surfaces of uterine and trophoblastic epithelia during embryo attachment in the cow. Anatomical Record 204 315–322. (10.1002/ar.1092040404) 7181136

[bib14] Haeger JD Hambruch N Dilly M Froehlich R & Pfarrer C 2011 Formation of bovine placental trophoblast spheroids. Cells, Tissues, Organs 193 274–284. (10.1159/000320544) 20975254

[bib15] Haeger JD Hambruch N & Pfarrer C 2016 The bovine placenta in vivo and in vitro. Theriogenology 86 306–312. (10.1016/j.theriogenology.2016.04.043) 27155733

[bib16] Halloran KM Stenhouse C Moses RM Kramer AC Sah N Seo H Lamarre SG Johnson GA Wu G & Bazer FW 2023 The ovine conceptus utilizes extracellular serine, glucose and fructose to generate formate via the one carbon metabolism pathway. Amino Acids 55 125–137. (10.1007/s00726-022-03212-x) 36383272

[bib17] Hoffman LH & Wooding FB 1993 Giant and binucleate trophoblast cells of mammals. Journal of Experimental Zoology 266 559–577. (10.1002/jez.1402660607) 8371098

[bib18] Hradecký P Mossman HW & Stott GG 1988 Comparative development of ruminant placentomes. Theriogenology 29 715–729. (10.1016/s0093-691x(8880016-5) 16726391

[bib19] Huttenlocker A & Horwitz AR 2011 Integrins in cell migration. Cold Spring Harbor Perspectives in Biology 3 a005074. (10.1101/cshperspect.a005074) 21885598 PMC3181029

[bib20] Johnson GA Bazer FW Seo H Burghardt RC Wu G Pohler KG & Cain JW 2022 Understanding placentation in ruminants: a review focusing on cows and sheep. Reproduction, Fertility and Development 36 93–111. (10.1071/RD23119) 38064193

[bib21] Johnson GA Seo H Bazer FW Wu G Kramer AC McLendon BA & Cain JW 2023 Metabolic pathways utilized by the porcine conceptus, uterus, and placenta. Molecular Reproduction and Development 90 673–683. (10.1002/mrd.23570) 35460118

[bib22] Klisch K Hecht W Pfarrer C Schuler G Hoffmann B & Leiser R 1999 DNA content and ploidy level of bovine placentomal trophoblast giant cells. Placenta 20 451–458. (10.1053/plac.1999.0402) 10419810

[bib23] Pohler KG Pereira MHC Lopes FR Lawrence JC Keisler DH Smith MF Vasconcelos JLM & Green JA 2016a Circulating concentrations of bovine pregnancy-associated glycoproteins and late embryonic mortality in lactating dairy herds. Journal of Dairy Science 99 1584–1594. (10.3168/jds.2015-10192) 26709163

[bib24] Pohler KG Peres RFG Green JA Graff H Martins T Vasconcelos JLM & Smith MF 2016b Use of bovine pregnancy-associated glycoproteins to predict late embryonic mortality in postpartum Nelore beef cows. Theriogenology 85 1652–1659. (10.1016/j.theriogenology.2016.01.026) 26928645

[bib25] Reese ST Pereira MHC Edwards JL Vasconcelos JLM & Pohler KG 2018 Pregnancy diagnosis in cattle using pregnancy associated flycoprotein concentration in circulation at day 24 of gestation. Theriogenology 106 178–185. (10.1016/j.theriogenology.2017.10.020) 29073542

[bib26] Sasser RG Ruder CA Ivani KA Butler JE & Hamilton WC 1986 Detection of pregnancy by radioimmunoassay of a novel pregnancy-specific protein in serum of cows and a profile of serum concentrations during gestation. Biology of Reproduction 35 936–942. (10.1095/biolreprod35.4.936) 3814705

[bib27] Seo H Bazer FW Burghardt RC & Johnson GA 2019 Immunohistochemical examination of trophoblast syncytialization during early placentation in sheep. International Journal of Molecular Sciences 20 4530–4543. (10.3390/ijms20184530) 31540219 PMC6769582

[bib28] Seo H Frank JW Burghardt RC Bazer FW & Johnson GA 2020 Integrins and OPN localize to adhesion complexes during placentation in sheep. Reproduction 160 521–532. (10.1530/REP-20-0273) 32668403

[bib29] Seo H Bazer FW & Johnson GA 2023 Early syncytialization of the ovine placenta revisited. In Syncytia: Origin, Structure, and Functions, 71st ed. Eds., Kloc M Uosef A. Springer Nature 127–142. (10.1007/978-3-031-37936-9_7) 37996676

[bib30] Thatcher WW Hansen PJ Gross TS Helmer SD Plante C & Bazer FW 1989 Antiluteolytic effects of bovine trophoblast protein-1. Journal of Reproduction and Fertility 37 91–99. 2810237

[bib31] Wallace RM Pohler KG Smith MF & Green JA 2015 Placental PAGs: gene origins, expression patterns, and use as markers of pregnancy. Reproduction 149 R115–R126. (10.1530/REP-14-0485) 25661256

[bib32] Wathes DC & Wooding FB 1980 An electron microscopic study of implantation in the cow. American Journal of Anatomy 159 285–306. (10.1002/aja.1001590305) 7211711

[bib33] Wimsatt WA 1951 Observations on the morphogenesis, cytochemistry, and significance of the binucleate giant cells of the placenta of ruminants. American Journal of Anatomy 89 233–281. (10.1002/aja.1000890204) 14894441

[bib34] Wooding FB 1982a The role of the binucleate cell in ruminant placental structure. Journal of Reproduction and Fertility 31 31–39. 6762432

[bib35] Wooding FB 1982b Structure and function of placental binucleate ('giant') cells. Bibliotheca Anatomica 22 134–139. 7126144

[bib36] Wooding FB 1984 Role of binucleate cells in fetomaternal cell fusion at implantation in the sheep. American Journal of Anatomy 170 233–250. (10.1002/aja.1001700208) 6465051

[bib37] Wooding FB 1992 Current topic: the synepitheliochorial placenta of ruminants: binucleate cell fusions and hormone production. Placenta 13 101–113. (10.1016/0143-4004(9290025-o) 1631024

[bib38] Wooding FBP 2022 The ruminant placental trophoblast binucleate cell: an evolutionary breakthrough. Biology of Reproduction 107 705–716. (10.1093/biolre/ioac107) 35594454 PMC9476219

[bib39] Wooding FBP & Burton G 2008 Synepitheliochorial placentation: ruminants (ewe and cow). In Comparative Placentation. Berlin, Heidelberg: Springer, pp. 133–167. (10.1007/978-3-540-78797-6_6)

