# Supplementary material for: Immunohistochemical examination of the uteroplacental interface of cows on days 21, 31, 40, and 67 of gestation
Source: Reproduction. 2024 Jan 29;167(2):e230444. doi: 10.1530/REP-23-0444 (PMC10895283; doi:10.1530/REP-23-0444)

Supplemental Figure 1.

Immunofluorescence staining for serine hydroxymethyltransferase 2 [SHMT2; green, stains some mononucleate trophoblast cells and trophoblast giant cells (TGCs) in the trophoblast layer, extraembryonic endoderm (En) cells and luminal epithelial (LE) cells] and E-cadherin (red) at the uterine-placental interface on day 20 **(A)** and day 31 **(B)**. SHMT2 is expressed by mononucleate cells (arrow heads) at the leading front of placental invasion into the caruncular stroma on day 40 **(C)**. The SHMT2-positive mononucleate cells (arrow head) have expanded to become the epithelial surface separating the caruncular stroma from the cotyledonary stroma within the placentome on day 67 **(D)**. The width of field for **Panels A-D** is 448 µm. Car, caruncular stroma; Tr, trophoblast; D, day; P, pregnancy.

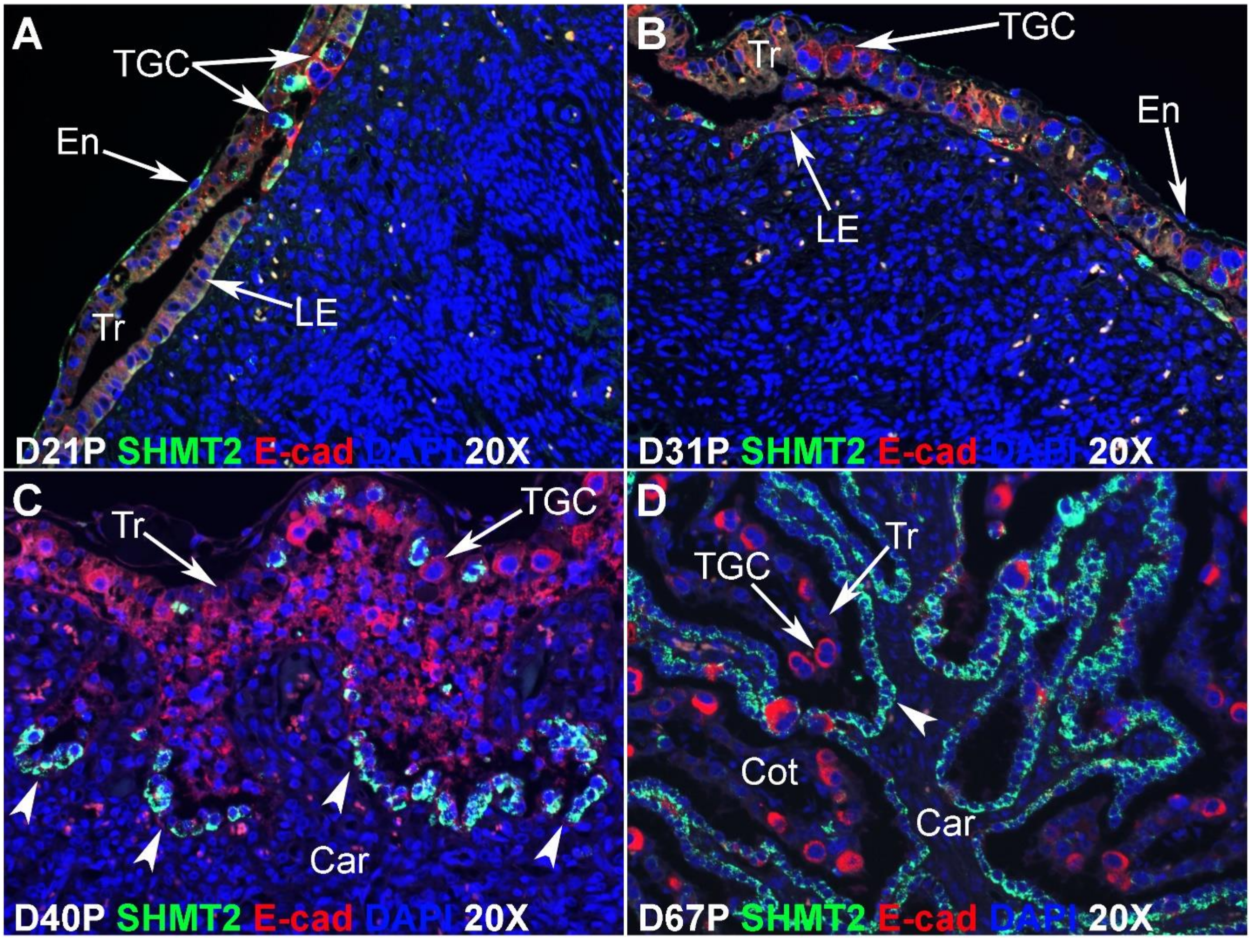

Supplement: Supplemental Figure 1. Immunofluorescence staining for serine hydroxymethyltransferase 2 [SHMT2; green, stains some mononucleate trophoblast cells and trophoblast giant cells (TGCs) in the trophoblast layer, extraembryonic endoderm (En) cells and luminal epithelial (LE) cells] and E-cadherin (red) a [file supplementary_figure_1.pdf]
